# Post-infection functional gastrointestinal disorders following coronavirus disease-19: a prospective follow-up cohort study

**DOI:** 10.1186/s12879-023-08401-x

**Published:** 2023-06-21

**Authors:** Daya Zhang, Chen Chen, Yunqian Xie, Fan Zeng, Shiju Chen, Runxiang Chen, Xiaodong Zhang, Shimei Huang, Da Li, Feihu Bai

**Affiliations:** 1 Graduate School, Hainan Medical University, Haikou, 571199 China; 2 Department of Gastroenterology, The Second Affiliated Hospital of Hainan Medical University, Yehai Avenue, #368, Longhua District, Haikou, 570216 Hainan Province China; 3 The Gastroenterology Clinical Medical Center of Hainan Province, Haikou, 570216 China

**Keywords:** Coronavirus disease (COVID-19), Functional Gastrointestinal Disorders (FGID), Post infection-irritable bowel syndrome (PI-IBS), Gastrointestinal(GI) symptoms, Rome IV, Rome III, Risk factors

## Abstract

**Background:**

Acute gastrointestinal infections can lead to post-infectious irritable bowel syndrome (PI-IBS). Moreover, coronavirus disease (COVID-19) is related to long-term gastrointestinal sequelae. In this study, the frequency, disease spectrum, and risk factors for post-infection functional gastrointestinal disease (PI-FGID) in COVID-19 patients and healthy controls were prospectively examined.

**Methods:**

Validated Rome III and Rome IV questionnaires and limited objective assessment were used to assess the incidence of PI-FGID in 190 COVID-19 patients, and 160 healthy controls prospectively followed for 1, 3, and 6 months.

**Results:**

Six(3.2%), 1(0.5%), 3(1.6%), 5(2.6%), 6(3.2%)COVID-19 patients had diarrhea, abdominal pain, constipation, dyspepsia and their overlap at 1 month, respectively, while 4(2.1%), 1(0.5%), 4(2.1%), 4(2.1%), and 6(3.2%)COVID-19 patients had diarrhea, abdominal pain, constipation, dyspepsia and their overlap at three months, respectively. Furthermore, 2(1.3%), 4(2.5%), and 3(1.9%)healthy controls developed constipation, dyspepsia, and their overlap at one month, respectively (*P* = 0.193), while 2(1.3%), 4(2.5%), and 2(1.3%)healthy controls developed constipation, dyspepsia and their overlap at three months, respectively (*P* = 0.286). FGIDs incidence was higher among COVID-19 patients(8.9%) than in healthy controls(3.1%) at 6-month follow-up (*P* = 0.025). Moreover, 7 (3.7%), 5 (2.6%), 3 (1.6%), and 2 (1.1%) COVID-19 patients developed IBS, functional dyspepsia(FD), functional diarrhea(FDr), functional constipation(FC)at six months, respectively, while only 2 (1.3%) and 3 (1.9%) healthy controls developed IBS and FD at six months, respectively. Notably, gastrointestinal(GI)symptoms at onset were the independent risk factors for post-COVID-19 FGIDs at six months.

**Conclusions:**

COVID-19 increases new-onset PI-FGID at six months compared with healthy controls. GI symptom at the onset of COVID-19 is an independent risk factor for post-COVID-19 FGIDs.

**Supplementary Information:**

The online version contains supplementary material available at 10.1186/s12879-023-08401-x.

## Introduction

Functional gastrointestinal disease (FGID) is a group of digestive disorders, including functional constipation (FC), functional diarrhea (FDr), irritable bowel syndrome (IBS), and functional dyspepsia (FD), and have a prevalence of 40.3% worldwide [1, 2]. However, the pathophysiology of FGID is unknown and may be multidimensional, involving genetic predisposition, eating factors, intestinal dysregulation, immune activation, altered intestinal permeability, and misregulation of the gut-brain axis [3].

Gastrointestinal(GI) bacterial, protozoal, and viral infections are risk factors for post-infectious FGID (PI-FGID) [4, 5]. In addition, PI-FGIDs are associated with intestinal microecology, including small intestinal bacterial overgrowth (SIBO), altered intestinal permeability, and low levels of persistent immune activation [6].

A severe acute respiratory syndrome coronavirus 2 (SARS-CoV-2) emerged in Wuhan, China, in 2019 [7], infecting over 300 million people worldwide, and thus is a major global public health concern [8]. SARS-CoV-2 can cause GI manifestations, such as abdominal pain, diarrhea, nausea, and vomiting, because of the presence of angiotensin-converting enzyme-2 (ACE-2) receptors in the GI epithelium [9–11]. A studie have reported that nearly half of Coronavirus disease (COVID-19) patients experience GI discomfort [12]. Also, the incidence of IBS and FD is significantly higher in COVID-19 patients than in healthy population [12, 13].

Nonetheless, the frequency, spectrum, and risk factors for the occurrence of PI-FGID after COVID-19 should be investigated. Previous studies on PI-FGID after COVID-19 lacked prospective controls. In addition, GI symptoms at follow-up in previous studies were mainly self-reported, and no validated questionnaire was used. In this study, the frequency, spectrum, and risk factors of PI-FGID following COVID-19 were prospectively investigated and compared with healthy controls.

## Methods

### Study design

This prospective cohort study was conducted on adults between July 2022 and February 2023. At the time of the study, China was tough on COVID-19 dominated by Omicron [14, 15], a variant of SARS-CoV-2 and all people were in a population-wide COVID screening in hospital or community with regular universal reverse transcription polymerase chain reaction (RT-PCR) test. All suspicious COVID persons even for asymptomatic infection were referred to local hospitals or large square cabin hospitals for mandatory quarantine. All suspicious infected persons undergoing a diagnostic procedure, such as a nasopharyngeal polymerase chain reaction (PCR) test or chest radiograph to show their lung involvement, had a definite diagnosis of coronavirus infection by their physician. Patients with COVID−19 are included in the study according to inclusion or exclusion criteria as soon as the diagnosis of COVID−19 is confirmed. Gastroenteroscopy including endoscopic biopsy, blood sampling(Blood count, white blood cell count, CPR, PCT, IL−6, IgG and IgM antibody tests for viruses) and other tests (X-rays and ultrasound, Urine routine, stool routine) were needed to exclude other bacterial and viral infections in the whole body or any part of the body or acute gastroenteritis (AG) that could cause GI symptoms. The lag time from diagnosis of COVID−19 to study inclusion is no more than 1 day. All patients received the national protocol of standard treatment without changes.

It consisted of 2 cohorts, a case group that included COVID-19 patients consecutively recruited from Fangcai Hospital, a dedicated COVID care center, in Haikou of Hainan Province, China. Healthy control group included COVID serology negative screeners at our hospital in the same time period. Participants were followed up either outpatient physical or over the telephone at 1, 3, and 6 months using validated Rome III and Rome IV questionnaire and limited objective assessment [1]. The follow-up period from July-August 2022 to February 2023 was more than 6 months. The data from case groups were compared with the 6-month follow-up data on the development of FGID in an age- and sex-matched cohort of healthy subjects. In addition, subjects who met the various diagnostic criteria for FGID in Rome at 6 months of follow-up were advised to come to the clinic for further examination by laboratory and endoscopic methods as well as clinical indications to exclude some diseases such as gastroparesis, SIBO, microscopic colitis, etc. Clinical indicators include duration and frequency of diarrhea, consistency of stool and presence of blood, history of vomiting, fever, cramping abdominal pain and weight loss. The included COVID-19 patients and healthy controls were tested for COVID-19 at 3 and 6 months to exclude COVID-19 reinfection. The questionnaires also included co-morbidities (Hypertension or diabetes), anxiety, irregular diet, sleep quality, and regular exercise sections.

Inclusion criteria of case group were as follows: (1) age between 18 and 85 years old, having a definitive diagnosis of coronavirus with different severity [16, 17] (mild and moderate) using biochemical test data (such as COVID19-SARS-CoV-2 positive, and anti-SARS-CoV-19 IgG and IgM negative) or computed tomography scan of chest and observation of lung involvement;(2) no previous history of clinical confirmation of the diagnosis of any FGID, no GI tumors, reflux esophagitis, ulcerative colitis or other GI diseases, no co-morbidities (Hypertension or diabetes), no history of abdominal surgery, and no any recurrent baseline symptoms of FGID or GI symptoms such as diarrhea, constipation, or abdominal pain; (3) blood sampling, and other tests were needed to exclude AG, bacterial and viral infections;(4) normal findings of gastroscopy and other laboratory tests from the last 6 months of medical examination were required. Healthy control group had no history of COVID-19 or FGID. Biochemical test data (such as COVID19-SARS-CoV-2 negative, and anti-SARS-CoV-19 IgG and IgM negative) are required and the rest of the criteria were the same as for the case group.

The objectives of this study were explained in detail to all participants, and also, partici-pating in this study was fully conscious and based on their desire. Further, written informed consent was obtained from each participant. Meanwhile, Each participant provided a written or electronic informed consent form and the study was approved by the institutional ethics committee of the Second Hospital of Hainan Medical University (reference number: LW2022270).

### Definitions

RT-PCR was used to detect SARS-CoV-2 in nasopharyngeal and oropharyngeal samples of subjects [18]. The diagnosis of IBS by the Rome IV diagnostic criteria has changed significantly, the new diagnostic criteria have increased the frequency required to diagnose IBS, and the incidence of IBS has decreased [1]. We used Rome III rather than the most recently described Rome IV criteria as the latter is 50% less sensitive to diagnose IBS [1]. Other types of FGID were diagnosed based on the Rome IV criteria [1].

Dyspepsia refer to postprandial discomfort syndrome, which often manifests itself as postprandial fullness and early satiety (inability to complete a normal meal). Dyspepsia means the same as Functional dyspepsia, and refers only to postprandial discomfort syndrome.

AG was defined as the presence of at least two of the followings: (i) diarrhea, (ii) vomiting, (iii) fever, and (iv) stool culture isolating entero-pathogens [6]. Stool sample of each COVID-19 patient was examined under a microscope for detection of pus cell, RBC or parasites. All suspected stool samples were cultured for Vibrio cholerae, Salmonella, Shigella, Campylobacter and Aeromonas using standard techniques to identify the pathogenic strains.

Patients who showed signs of organ failure (such as persistent oliguria, severe respiratory distress) or required ventilator support or admission to an intensive care unit during treatment for COVID-19 infection were defined as having severe COVID-19. The severity of the COVID-19 [16, 17, 19] was assessed as described: (i) critical (required ventilator), (ii) severe (needed oxygen), (iii) moderate (though pneumonia present, did not require oxygen), and (iv) mild (only upper respiratory symptoms). Those without symptoms at the time of diagnosis of COVID-19 were classified as asymptomatic as categorical variables (yes or no).

Living with frequent (> 5 times/month) infrequent meals and irregular meal times was defined as an irregular diet. The Pittsburgh Sleep Quality Index (PSQI) [20] was used to investigate the sleep quality of patients. The total score of PSQI ranges from 0 to 21, where 7 is the threshold value of sleep quality problem, and the higher score above 7 indicates the poorer sleep quality of the patients, while the opposite indicates the better sleep quality of the patients. Regular exercise is defined by the latest World Health Organization (WHO) Guidelines for Exercise and Sedentary Behavior 2020 [21]. Adults should engage in at least 150–300 min of moderate-intensity aerobic exercise per week, or at least 75–150 min of high-intensity aerobic activity, or an equivalent combination of moderate-intensity and high-intensity exercise; Older adults should have at least 150–300 min of moderate intensity aerobic exercise per week, or at least 75–150 min of high intensity aerobic exercise, or an equivalent combination of moderate and high intensity exercise. The Hamilton Anxiety Scale [22] was used to evaluate the psychological status of the case group, and possible anxiety was considered when the scale score was > 7.

### Statistical analysis

#### Sample size calculation

**Fig. 1:**
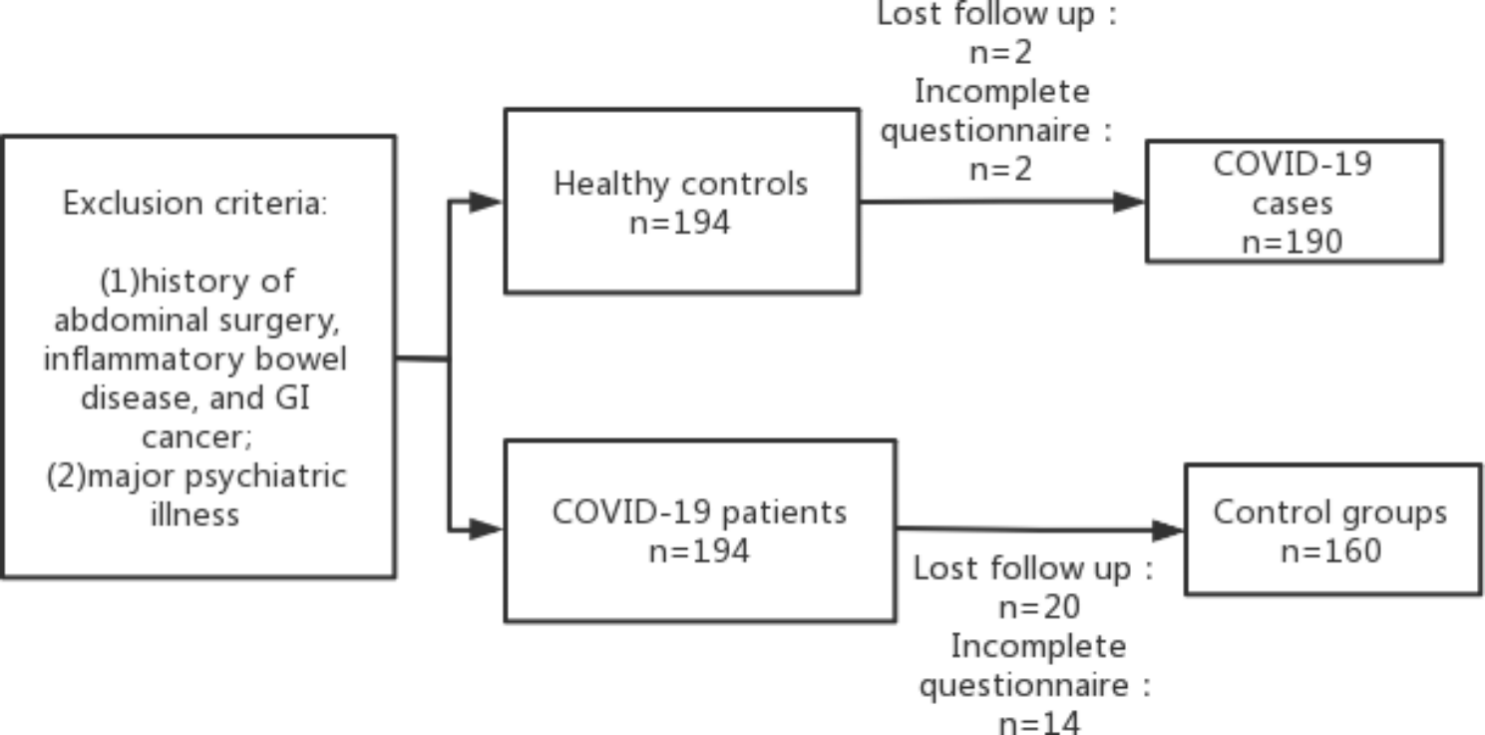
Flowchart of this study

In this study, the sample size was calculated as 90% power, 99% confidence interval (bilateral) based on previous studies [12, 23, 24] that showed that the mean incidence of FGIDs after acute gastroenteritis is about 21% and a Chinese study that showed the incidence of PI-FGIDs in controls of 8.2% [25]. A total of 155 COVID-19 patients and 155 healthy controls were required in this study, while 194 COVID-19 patients and 194 controls were enrolled since about 10–20% loss could occur at follow-up. Four of 194 COVID-19 patients and 34 of 194 healthy controls were excluded because they had inadequate documentation or were lost during follow-up. Finally, 190 COVID-19 patients and 160 healthy controls were analyzed (Fig. 1).

### Data collection and analysis

SPSS (version 26; SPSS Inc, Chicago, IL) was used for all statistical analyses. Categorical data were presented as proportions, while continuous data were expressed as mean ± standard deviation (SD) or median and range or interquartile range (IQR). Categorical variables were analyzed using χ2 test with Yates correction, where applicable. Parametric and nonparametric continuous data were analyzed using unpaired t-test and Mann-Whitney U test, respectively. Multivariate analysis was performed using stepwise logistic regression. A two-tailed P value < 0.05 was considered significant.

## Results

### Baseline demographics and clinical profile

The age [44.5 (36–54) years vs. 44.5 (32–57) years; *P* = 0.672] and gender [96/190 (50.5%) vs. 79/160 (49.4%) males; *P* = 0.830] were not significantly different between the COVID-19 patients and healthy controls. A total of 69 (36.3%) COVID patients were asymptomatic. The baseline characteristics of the patients are presented in Table 1.

**Table 1:** Comparison of the COVID-19 cases and control groups

Parameter	COVID-19 cases (190)	Control group (160)	χ2/Z	*P*
Age (year, median, IQR)	44.5(36–54)★	44.5(32–57)	-0.423	0.672
Gender			0.046	0.830
Male	96(50.5%)	79(49.4%)		
Female	94(49.5%)	81(50.6%)		
Severity of COVID-19				
Asymptomatic	69 (36.3%)	/		
symptomatic	121(63.7%)	/		
GI symptoms during infection	30(15.8%)	/		
Diarrhea only	17(8.9%)	/		
Abdominal pain only	8(4.2%)	/		
Constipation only	7(3.7%)	/		
Dyspepsia only	10(5.3%)	/		
Overlap	9(4.7%)	/		
GI symptoms in the first month			6.876	0.193
Diarrhea only	6(3.2%)	0 (0.0%)		
Abdominal pain only	1(0.5%)	0 (0.0%)		
Constipation only	3(1.6%)	2(1.3%)		
Dyspepsia only	5(2.6%)	4(2.5%)		
Overlap	6(3.2%)	3(1.9%)		
None	169(88.9%)	151(94.4%)		
GI symptoms in the third month			5.842	0.286
Diarrhea only	4(2.1%)	0(0.0%)		
Abdominal pain only	1(0.5%)	0(0.0%)		
Constipation only	4(2.1%)	2(1.3%)		
Dyspepsia only	4(2.1%)	4(2.5%)		
Overlap	6(3.2%)	2(1.3%)		
None	171(90.0%)	152(95.0%)		
Incidence of PI-FGID	17 (8.9%)	5 (3.1%)	4.999	0.025
IBS	7 (3.7%)	2 (1.3%)	/	0.189
FD	5 (2.6%)	3 (1.9%)	/	0.732
FDr	3 (1.6%)	0 (0.0%)	/	0.253
FC	2 (1.1%)	0 (0.0%)	/	0.502

★ Mann-Whitney U test; COVID, coronavirus disease; IQR, interquartile range; GI, gastrointestinal; PI-FGID, post infection functional gastrointestinal disease; FC, functional constipation; FDr, functional diarrhea; IBS, irritable bowel syndrome; FD, functional dyspepsia

### Baseline gastrointestinal symptoms

Thirty(15.8%)of 190 COVID-19 patients had GI discomfort, including diarrhea (n = 17, 8.9%), abdominal pain (n = 8, 4.2%), constipation (n = 7, 3.7%), dyspepsia (n = 10, 5.3%) and overlap (n = 9, 4.7%)(Table 1).

### PI-FGID among COVID-19 patients and healthy controls

**Fig. 2:**
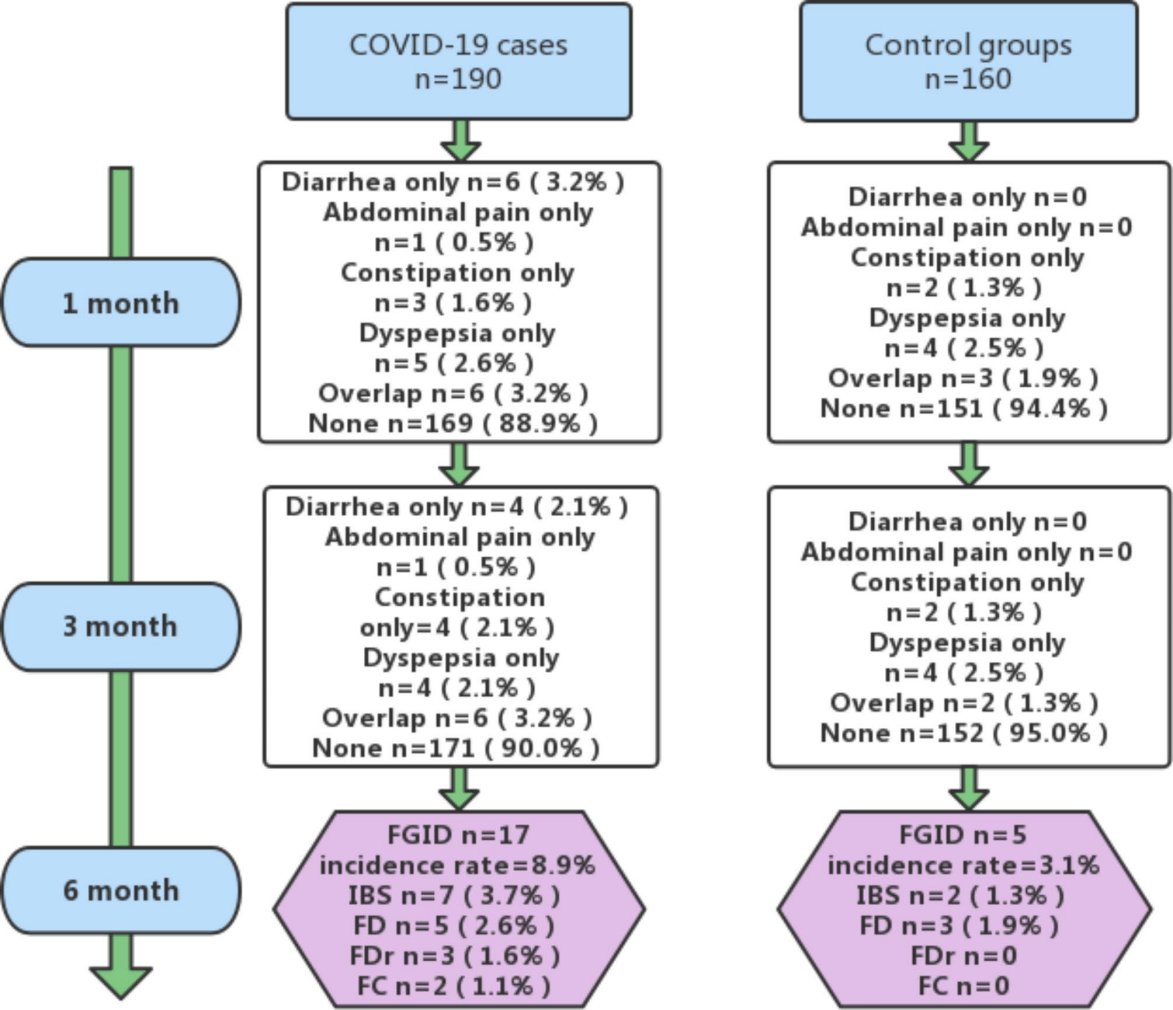
Comparison of the COVID-19 cases and control groups

Six(3.2%), 1(0.5%), 3(1.6%), 5(2.6%), and 6(3.2%) COVID-19 patients suffered from diarrhea, abdominal pain, constipation, dyspepsia and their overlap at one month, respectively while 4(2.1%), 1(0.5%), 4(2.1%), 4(2.1%), and 6(3.2%)COVID-19 patients suffered from diarrhea, abdominal pain, constipation, dyspepsia and their overlap at three months, respectively. Furthermore, 2(1.3%), 4(2.5%), and 3(1.9%)healthy controls developed constipation, dyspepsia, and their overlap at one month, respectively (*P* = 0.193) while 2(1.3%), 4(2.5%), and 2(1.3%)healthy controls developed constipation, dyspepsia and their overlap at three months, respectively (*P* = 0.286). FGIDs incidence was higher among COVID-19 (8.9%) patients than in healthy controls (3.1%) during 6-month follow-up (*P* = 0.025). Moreover, 7 (3.7%), 5 (2.6%), 3 (1.6%), and 2 (1.1%) COVID-19 patients developed IBS, FD, FDr, FC respectively, while only 2 (1.3%) and 3 (1.9%) healthy controls developed IBS and FD, respectively. The above results are shown in Fig. 2; Table 1.

### Prevalence of post-COVID-19-FGID in patients with and without GI symptoms at onset

Compared with healthy controls, COVID-19 patients with GI symptoms at onset had a higher frequency of GI symptoms at 1, 3, and 6 months (16/30 [53.3%] vs. 5/160 [3.1%]; P < 0.001); 14/30 [46.7%] vs. 5/160 [3.1%]; *P* < 0.001; 13/30 (43.3%) vs. 4/160 (2.5%); *P* < 0.001, respectively) (Table 2). Compared with asymptomatic patients, the symptomatic patients were more likely to suffer from diarrhea (5/30 [16.7%] vs. 1/160 [0.6%]; *P* < 0.001), dyspepsia (3/30 [10.0%] vs. 2/160 [1.3%; *P* = 0.029) and their overlap (5/30 [16.7%] vs. 1/160 [0.6%]; *P* < 0.001) at one month. Similarly, the symptomatic patients were more likely to suffer from diarrhea (3/30 [10.0%] vs. 1/160 [0.6%]; *P* = 0.013), dyspepsia (3/30 [10.0%] vs. 1/160 [0.6%; *P* = 0.013), and their overlap (5/30 [16.7%] vs. 1/160 [0.6%]; *P* < 0.001), compared with asymptomatic patients. At 6-month follow-up, the symptomatic patients were more likely to develop IBS (6/30 [20.0%] vs. 1/160 [0.6%]; *P* < 0.001) and FD (4/30 [13.3%] vs. 1/160 [0.6%]; *P* = 0.002) compared with asymptomatic patients. These results are shown in Table 2.

**Table 2:** Relationship between GI symptoms and consequences of post-COVID-19 GI

Parameters	GI symptoms (n = 30)	GI symptoms absent (n = 160)	χ2	*P* values
Presence of dyspeptic symptoms, and their overlap at 1 month			49.276	< 0.001
Diarrhea only	5(16.7%)	1(0.6%)	/	< 0.001
Abdominal pain only	1(3.3%)	0(0.0%)	/	0.158
Constipation only	2(6.7%)	1(0.6%)	/	0.065
Dyspepsia only	3(10.0%)	2(1.3%)	/	0.029
Overlap	5(16.7%)	1(0.6%)	/	< 0.001
None	14(46.7%)	155(96.9%)	59.773	< 0.001
Presence of dyspeptic symptoms, and their overlap at 3 months			41.269	< 0.001
Diarrhea only	3(10.0%)	1(0.6%)	/	0.013
Abdominal pain only	1(3.3%)	0(0.0%)	/	0.158
Constipation only	2(6.7%)	2(1.3%)	/	0.118
Dyspepsia only	3(10.0%)	1(0.6%)	/	0.013
Overlap	5(16.7%)	1(0.6%)	/	< 0.001
None	16(53.3%)	155(96.9%)	48.490	< 0.001
PI-FGID(IBS, FD, FDr and FC) at 6 months			38.863	< 0.001
IBS	6(20.0%)	1(0.6%)	21.544	< 0.001
FD	4(13.3%)	1(0.6%)	/	0.002
FDr	2(6.7%)	1(0.6%)	/	0.065
FC	1(3.3%)	1(0.6%)	/	0.292
None	17(56.7%)	156(97.5%)	46.814	< 0.001

COVID, coronavirus; GI, gastrointestinal; FC, functional constipation; FDr, functional diarrhea; IBS, irritable bowel syndrome; FD, functional dyspepsia; PI-FGID, post-infection functional gastrointestinal disease

### Risk factors of post-COVID-19 FGID at six months

Post-COVID-19 FGID at six months was related to GI symptoms at the onset or at 1 and 3 months, COVID-19 severity, and anxiety state after COVID-19 (Table 3). Multivariate analysis showed that GI symptom at the onset was an independent risk factor for post-COVID-19 FGID (Table 4).

**Table 3:** Risk Factors associated with PI-FGID

Parameters	FGID Present (n = 17)	No FGID (n = 173)	χ2/t	*p*
Age, mean(y)	39.71 ± 9.43	44.81 ± 12.85	0.291	0.113
Gender			0.044	0.835
male	9(52.9%)	87(50.3%)		
Female	8(47.1%)	86(49.7%)		
Presence of comorbidity	3 (17.6%)	20 (11.6%)	0.119	0.730
Severity of COVID-19			4.866	0.027
Asymptomatic	2(11.8%)	67(38.7%)		
Symptomatic	15(88.2%)	106(61.3%)		
GI symptoms during COVID-19			67.834	< 0.001
Yes	15 (88.2%)	15 (8.7%)		
No	15 (11.8%)	15 (91.3%)		
Presence dyspeptic symptoms, and their overlap at 1 month			97.877	< 0.001
Diarrhea only	3 (17.6%)	3 (1.1%)		
Abdominal pain only	1 (5.9%)	0 (0.0%)		
Constipation only	3 (17.6%)	0 (0.0%)		
Dyspepsia only	4 (23.5%)	1(0.6%)		
Overlap	6 (35.3%)	0 (0.0%)		
None	0 (0.0%)	169 (97.7%)		
Presence dyspeptic symptoms, and their overlap at 3 months			101.872	< 0.001
Diarrhea only	3 (17.6%)	1 (0.6%)		
Abdominal pain only	1 (5.9%)	0 (0.0%)		
Constipation only	3 (17.6%)	1 (0.6%)		
Dyspepsia only	4 (23.5%)	0 (0.0%)		
Overlap	6 (35.3%)	0 (0.0%)		
None	0 (0.0%)	171 (98.8%)		
Anxiety state after COVID-19			19.964	< 0.001
Yes	12(35.2%)	33(10.9%)		
No	5(29.4%)	140(80.9%)		
Irregular diet			1.191	0.275
Yes	3(17.6%)	12(6.9%)		
No	14(82.4%)	161(93.1%)		
Good sleep quality			1.907	0.167
Yes	3(17.6%)	59(34.1%)		
No	14(82.4%)	114(65.9%)		
Regular exercise				
Yes	3(17.6%)	72(41.6%)	3.723	0.054
No	14(82.4%)	101(58.4%)		

COVID, coronavirus; GI, gastrointestinal; PI-FGID, post infection functional gastrointestinal disease

**Table 4:** Multivariate analysis of Factors associated with PI-FGID

Parameters	Crude odds ratio (95% CI)	Crude *P* values	Adjusted odds ratio (95% CI)	Adjusted *P* values
Severity of COVID-19 (reference, asymptomatic)	4.741(1.051–21.390)	0.043	1.015(0.148–6.964)	0.988
Presence of GI symptoms (reference: no GI symptom)	79.000(16.476-378.799)	< 0.001	52.049(9.651-280.697)	< 0.001
Presence dyspeptic symptoms, and their overlap at 1 month (yes vs. no)	/	0.994	/	/
Presence dyspeptic symptoms, and their overlap at 3 months (yes vs. no)	/	0.994	/	/
Anxiety state after COVID-19	10.182(3.355–30.898)	< 0.001	3.502(0.885–13.855)	0.074

COVID, coronavirus; GI, gastrointestinal; PI-FGID, post infection functional gastrointestinal disease

## Discussion

COVID-19 pandemic has significantly changed lifestyles and cause stress worldwide. COVID-19 can affect post-recovery of gut function by resulting in underlying pathophysiological alterations, including dysbiosis, intestinal barrier disruption, intestinal inflammation, intestinal-pulmonary axis damage, immune dysregulation, and psychological stress [26]. However, only a few studies have reported the gastrointestinal sequelae of COVID-19 infection [27]. Besides, previous studies mainly studied IBS after COVID-19 infection, which only has a prevalence of 2.5-5.3% [27, 28]. This study investigated the prevalence of different FGIDs, including FC, FD, FDr and IBS.

This is not at all surprising that the COVID-19 increases new-onset PI-FGID at six months compared with healthy controls. COVID-19 also affects the GI tract because of the presence of ACE-2 receptors, the SARS-CoV-2 viral entry site, in the GI epithelium [26–30]. The detection of viral RNA in the stools of half of the patients [11], increased fecal calprotectin [12, 13], abnormal intestinal permeability [26], altered intestinal microbiota [26] and increased serotonin [16] may also indicate that SARS-CoV-2 not only infects the GI tract but may also lead to long-term GI consequences, such as FGID.

In this study, the symptomatic patients were at greater risk of PI-FGID than asymptomatic infected patients. Diarrhea during the initial period of infection until long after the infection was the most common GI symptom among the infected patients, similar to the findings of physicians who studied the initial GI symptoms among infected patients in Wuhan, China [12]. A meta-analysis with 4243 SARS-CoV-2 infected patients revealed that severely symptomatic infected patients are more prone to have GI symptoms [13]. A study identified SARS-CoV-2 in the stool of 52.4% of infected patients who also had GI symptoms and in only the stool of only 39.1% of the infected patients without GI symptoms [31]. In this study, Rome III questionnaire results showed that the incidence of IBS was not significantly different between the post-COVID cohort and healthy controls. Nonetheless, these observations should be cautiously interpreted since the study had a small number of post-COVID-19 FGID patients.

Psychological stress can trigger FGID development. Anxiety can predict the onset of FGID since about 23.65% of highly stressed stress individuals have at least one GI symptom, and about 31.1% of highly stress individuals have three or more GI symptoms during high stress [32]. In this study, anxiety or depression was not positively associated with the prevalence of post-COVID-19 FGID. PI-FGID, such as IBS and FD, are common among younger age and PI-FGID was more prevalent in women compared to men [1, 33]. In this study, age and gender were not significantly associated with the prevalence of PI-FGID, which may be related to the small sample of our study population and the fact that the study subjects were novel coronavirus-infected individuals.

The importance of prevention of COVID-19 not only has short-term but also long-term benefit. Recognizing the prevalence of GI sequelae associated with COVID-19 has important implications for clinical strategies and choices in patient care, and for giving patients more attention to GI sequelae during follow-up, as well as for the rational allocation of nursing and medical resources.

However, this study has some limitations. First, the study was a single-center cohort study with a small sample size, especially for the PI-FGID COVID-19 and results may not be generalizable. Second, it is also important to mention that the assessment of symptoms was based on questionnaires and limited objective assessment, so recall bias may be present. Third, different mutant strains of the COVID-19 exhibit differences in GI symptoms and severity in different regions of the world. Omicron, a variant of SARS-CoV-2, was first identified in 2021 [14] and spread rapidly worldwide by 2022 [15]. The prevalent strain in China at the time of our study was Omicron, and the representativeness of the results was limited. Fourth, there is also a possibility of COVID-19 infection (asymptomatic) in both cases and healthy group between the study follow-up that can create further bias. Fifth, we did not integrate the 3-month and 6-month COVID-19 reinfections into one study visit, because this would increase the follow-up time. Besides, as follow-up time increases, the COVID-19 trend are released, the infection rate will increase and it will be very difficult to find healthy controls. I acknowledge that it would be better to integrate it into one study visit, and we will continue to study this in the future including following two areas: (1) are the incidence of FGID increased and symptoms of FGID worsen after reinfection? (2) healthy controls who were previously negative for COVID-19 and became positive for infection after 3 or 6 months were included in the case group and followed up again, which will greatly increase the sample size of the case group and better validate the results. Last, the absence of psychological data preceeding the infection also represent limitations of the study.

In conclusion, COVID-19 individuals with GI symptoms may develop PI-FGID during the 6-month follow-up period. GI symptom at the onset of COVID-19 is an independent risk factor for post-COVID-19 FGIDs. Of course, the relationship between COVID-19 and FGID needs to be further demonstrated by more large sample and multicenter studies, and the mechanism of FGID caused by COVID-19 also needs to be further explored by scholars.

## Electronic supplementary material

Below is the link to the electronic supplementary material.

Supplementary Material 1

Supplementary Material 2

## Data Availability

The datasets generated and analyzed in this study are available from the corresponding author upon reasonable request.

## Abbreviations

Coronavirus disease: COVID-19
PI-IBS: Post-infectious irritable bowel syndrome
PI-FGID: Post-infection functional gastrointestinal disease
FC: Functional constipation
FDr: Functional diarrhea
IBS: Irritable bowel syndrome
FD: Functional dyspepsia
GI: Gastrointestinal
SIBO: Small intestinal bacterial overgrowth
SARS-CoV-2: Acute respiratory syndrome coronavirus 2
ACE-2: Angiotensin-converting enzyme-2
RT-PCR: Reverse transcriptase-polymerase chain reaction
5-HT: 5-hydroxytryptamine
