# Supplementary material for: Post-infection functional gastrointestinal disorders following coronavirus disease-19: a prospective follow-up cohort study
Source: BMC Infect Dis. 2023 Jun 21;23:422. doi: 10.1186/s12879-023-08401-x (PMC10286442; doi:10.1186/s12879-023-08401-x)
Supplement: Supplementary file 1 — Supplementary Material 1 [file 12879_2023_8401_MOESM1_ESM.docx]

**Pittsburgh Sleep Quality Index Scale and Scoring Criteria**

Name( ) Gender ( ) Age ( ) Date of examination ( ) ID ( )

The following questions are about your sleep in the last month. Please select the answer that best matches your actual situation in the last month. Please answer the following questions.

1. In the past month, you usually went to bed at night ( ) o'clock.

2. In the past month, it usually took ( ) minutes to go to bed and fall asleep.

3. In the last month, you usually get up at ( ) o'clock in the morning.

4. In the last 1 month, the actual number of hours of sleep per night is usually ( ) (not equal to the time spent in bed).

Please choose the most appropriate answer to the following questions.

5. In the past 1 month, you have been troubled by the following conditions that affect your sleep.

a. Difficulty in falling asleep (can't fall asleep within 30 minutes) (1) None (2) <1 time/week (3) 1-2 times/week (4) >3 times/week

b. Waking up easily or early at night (1) None (2) <1 time/week (3) 1-2 times/week (4) >3 times/week

c. Nighttime visits to the toilet (1) None (2) <1 time/week (3) 1-2 times/week (4) >3 times/week

d. Poor breathing (1) None (2) <1 time/week (3) 1-2 times/week (4) >3 times/week

e. High cough or snoring (1) None (2) <1 time/week (3) 1-2 times/week (4) >3 times/week

f. Feeling cold (1) None (2) <1 time/week (3) 1-2 times/week (4) >3 times/week

g. Feeling hot (1) None (2) <1 time/week (3) 1-2 times/week (4) >3 times/week

h. Nightmares (1) None (2) <1 time/week (3) 1-2 times/week (4) >3 times/week

i. Pain and discomfort (1) None (2) <1 time/week (3) 1-2 times/week (4) >3 times/week

j. Other things that affect sleep (1) None (2)<1 time/week (3) 1-2 times/week (4)>3 times/week

If yes, please specify.

6. In the past month, in general, do you think your sleep quality is (1) very good (2) good (3) poor (4) very poor?

7. In the past month, you have used medication to induce sleep (1) None (2) Less than 1 time/week (3) 1-2 times/week (4) >3 times/week

8. In the past month, do you often feel sleepy? (1) No (2) <1 time/week (3) 1-2 times/week (4) >3 times/week

9. In the past month, do you have little energy to do things (1) No (2) Occasionally (3) Sometimes (4) Often

Sleep quality score ( ) , sleep time score ( ) , sleep duration score ( ) , sleep efficiency score ( ) , sleep disorder score ( ) , hypnotic medication score ( ) , daytime dysfunction score ( )

The meaning of each component and the scoring method are as follows.

A Sleep quality: 1 point for good, 2 points for poor, and 3 points for very poor according to the response to entry 6.

B Sleep time

1. the scoring of entry 2: 15 points is 0, 16-30 points is 1, 31-60 points is 2, 60 points is 3.

2. the scoring of entry 5a: 15 points is 0, 16-30 points is 1, 31-60 points is 2, 60 points is 3, the score of entry 5a is 0 points for none, 1 point for <1 week/time, 2 points for 1~2 weeks/time, and 3 points for ≧3 weeks/time.

3. The cumulative score of entries 2 and 5a, if the cumulative score is 0, 0 points, 1~2, 1 point, 3~4, 2 points, 5~6, 3 points

C Sleep time

According to the response score of entry 4, >7 hours is scored as 0, 6~7 is scored as 1, 5~6 is scored as 2, and <5 hours is scored as 3.

D Sleep efficiency

1. bedtime = entry 3 (wake up time) - entry 1 (bedtime)

2. sleep efficiency = entry 4 (sleep time" bed time X 100%)

3. Component D scoring position, sleep efficiency > 85% scores 0, 75-84% scores 1, 65-74% + 2, < 65% scores 3.

E sleep disorder

Based on the scoring of entries 5b to 5j, 0 points for none, 1 point for < 1 week/time, 2 points for 1 to 2 weeks/time, and 3 points for ≧ 3 weeks/time. The cumulative score of entries 5b to 5j, if the cumulative score is 0, the component E is 0, 1 to 9 is 1, 10 to 18 is 2, 19 to 27 is 3.

F Hypnotic drugs

According to the response score of entry 7, 0 points for none, 1 point for <1 week/time, 2 points for 1-2 weeks/time, and 3 points for ≧3 weeks/time.

G Daytime dysfunction

1. Scored according to the response to entry 7, 0 points for none, 1 point for <1 week/time, 2 points for 1 to 2 weeks/time, and 3 points for >3 weeks/time.

2. The score based on the response to entry 7 is 0 points for none, 1 point for occasional, 2 points for occasional, and 3 points for frequent.

3. Accumulate the scores of entries 8 and 9. If the accrued score is 0, then component G is scored as 0, 1 to 2 is scored as 1, 3 to 4 is scored as 2, and 5 to 6 is scored as 3.

Total PSQI score = Component A + Component B + Component C + Component D + Component E + Component F + Component G

Evaluation level:

0-5 points sleep quality is very good

6-10 points sleep quality is okay

11-15 points average sleep quality

PSQI total score ( )
