# Supplementary material for: Post-infection functional gastrointestinal disorders following coronavirus disease-19: a prospective follow-up cohort study
Source: BMC Infect Dis. 2023 Jun 21;23:422. doi: 10.1186/s12879-023-08401-x (PMC10286442; doi:10.1186/s12879-023-08401-x)
Supplement: Supplementary file 2 — Supplementary Material 2 [file 12879_2023_8401_MOESM2_ESM.docx]

**Investigation of functional gastrointestinal disease**

Please note that only COVID-19 infected individuals are required to answer question 6 and 33, and questions10-24 are only required if the corresponding symptoms are present.

1. name:
2. gender:
3. age:
4. Telephone number：
5. Have you ever been infected with COVID-19:

○Yes

○No (Please present your RT-PCR test results for the past 1 year to the staff)

6. Only COVID-19 infected patients need to answer: What are the symptoms of your infection during the infection? [Single or multiple choice].

○No obvious symptoms

○Symptoms, fever, cough, malaise, etc. but not serious

○Symptoms, in addition to fever, cough and other symptoms there are also signs of organ failure (persistent oliguria, severe hypoxemia, severe respiratory distress, etc.)

○Symptoms, admitted to intensive care unit during treatment due to serious illness

| 7. Do you have the following symptoms repeatedly in your life now,If you have symptoms, please fill in the average frequency of symptoms in the last 3 months [Single or multiple choice] |
| --- |
| □ Postprandial fullness (Must answer question 10-13) Frequency:○<1 time/week ○1-2 times/week ○3 times/week ○>3 times/week |
| □ Early satiety (can't finish normal amount of food) (must answer question 10-13) Frequency: ○<1 time/week ○1 -2 times/week ○3 times/week ○>3 times/week |
| □ Abdominal pain (must answer questions 14-18) Frequency: ○<1 time/week ○1 -2 times/week ○3 times/week ○>3 times/week |
| □ straining to defecate, feeling of dry stool or incomplete defecation, feeling of anal obstruction (must answer questions 19-21) Frequency: ○<1 time/week ○1 -2 times/week ○3 times/week ○>3 times/week |
| □ Diarrhea (must answer questions 22-24) Frequency:○<1 time/week ○1 -2 times/week ○3 times/week ○>3 times/week |
| □ Bloating or abdominal bulging Frequency:○<1 time/week ○1 -2 times/week ○3 times/week ○>3 times/week |
| □ Other Gastrointestinal Discomfort _________________ |
| □ No gastrointestinal symptoms |

8. Any previous diagnosis of inflammatory bowel disease, gastrointestinal malignancy or history of abdominal related surgery, upper gastrointestinal bleeding (please answer yes if one of these)[Single-choice]

| ○Yes ○No |
| --- |

9. any previous gastroscopy and colonoscopy [Single-choice]

| ○Yes (Please fill in the test result: normal/abnormal and disease name) _________________  ○Never done  If you have symptoms of postprandial fullness and early satiety, please answer questions 10-13:  10.When did postprandial fullness and early satiety start to appear [Single-choice]:  ○Years or even decades ago  ○6 months ago  ○4-6 months  ○1-3 months  ○This 1 month  11.Does postprandial fullness interfere with your daily activities and cause you distress [Single-choice]:  ○Yes ○No  12.Does early satiety often result in your inability to complete a normal meal [Single-choice]:  ○Yes ○No   1. Are the symptoms of postprandial fullness and early satiety relieved after defecation or farting?[Single-choice]   ○Yes ○No |
| --- |

If you have symptoms of abdominal pain you must answer questions 14-18

14. When did the abdominal pain start to appear[Single-choice]:

○ Years or even decades ago

○6 months ago

○4-6 months

○1-3 months

○This 1 month

15. Are the following concomitant symptoms present during the onset of abdominal pain: [Single or multiple choice]

| ○Abdominal pain is associated with defecation (such as abdominal pain is relieved after defecation). |
| --- |
| ○Attacks of abdominal pain accompanied by changes in the frequency of bowel movements (e.g., increased or decreased frequency of bowel movements)  ○Episodes of abdominal pain accompanied by changes in the shape of the stool(e.g., irregular or hard stools)  ○No significant relationship between abdominal pain and defecation  16. Your symptoms belong to which of the following subtypes [Single-choice]   \| ○Constipation type: hard and lumpy stools accounted for >25% of stool volume \| \| --- \| \| ○Diarrhea type: loose stools and watery stools accounted for >25% of the stool volume \| \| ○Mixed type: dilute and watery stools accounted for >25% of stools and hard and lumpy stools accounted for >25% of stool volume \| \| ○Undefined: the above three are not in line  17. Whether abdominal pain is related to eating [Single-choice]:  ○Yes, pain attacks or worsens on an empty stomach  ○Yes, pain attacks or worsens after eating  ○No, there was no significant correlation between pain and eating \| |

18. Is abdominal pain relieved after taking stomach medication (e.g., Omeprazole) [Single-choice]:

○Yes, pain relief after medication

○No, no significant relief after medication

○No medication was used

If you have difficulty in defecation, constipation symptoms must answer questions 19-21

19. Does the following currently exist? [Single or multiple choice]

| □ More than 25% of the bowel movements were strained |
| --- |
| □ More than 25% of the bowel movements were dry (type 1 and 2 in the chart below) |
| □More than 25% of the bowel movements were incomplete |
| □ More than 25% of bowel movements have a feeling of anal obstruction |
| □ More than 25% of bowel movements require assistance (e.g., finger assistance, pelvic floor assistance) |
| □ Spontaneous bowel movements less than 3 times per week |
| □Very few loose stools when laxatives (e.g., lactulose) are not used  □ None of the above match  20. When did the above abnormal bowel movements first appear? [Single-choice]:  ○Years or even decades ago  ○6 months ago  ○4-6 months  ○1-3 months  ○This 1 month  21.Any long-term oral pain medication (e.g. codeine, morphine)[Single-choice]:  ○Yes ○No |

If you have symptoms of diarrhea you must answer questions 22-24

22.When did the diarrhea symptoms start to appear? [Single-choice]:

○Years or even decades ago

○6 months ago

○4-6 months

○1-3 months

○This 1 month

23.Do your symptoms match: 25% or more watery or loose stools (as shown below) [Single-choice]

| ○Fully conform ○Not quite conform  24.Is diarrhea accompanied by significant abdominal pain and bloating [Single-choice]  ○Yes ○No  25. Did you visit the hospital for the above-mentioned gastrointestinal discomfort [Single-choice]:  ○No  ○Yes and was diagnosed with _________________ |
| --- |

Life Questions:

26. The average frequency of bowel movements in a day is [Single-choice]:

○>3 times/day ○2-3 times/day ○1 time/day ○2-4 days/time

27. Are gastrointestinal symptoms causing you serious distress in your life now？ [Single-choice]:

○No symptoms, so it doesn't matter

○Symptoms but not affecting life

○Have symptoms that seriously affect life

28. the regularity of eating habits in life [Single-choice]

| ○Yes, eating three meals on time  ○No, often missing meals or irregular meal times (more than 5 times/month) |
| --- |

29. Cumulative number of hours of exercise per week [Single-choice]

| ○More than 4 hours  ○2 to 4 hours  ○1 to 2 hours  ○Less than 1 hour |
| --- |

30. recent sleep status [Single-choice]

| ○Good  ○General  ○Difficulty sleeping  ○Shallow sleep |  |
| --- | --- |

31. How much sleep do you get each day? [Single-choice]

| ○≥9 hours  ○7-9 hours  ○4-6 hours |
| --- |

32. Do you often feel depressed, anxious, or nervous in your daily life [Single-choice]

| ○Never  ○Sometimes (less than 4 times a month)  ○Frequently (more than 4 times a month)  33. COVID-19 infected patients need to answer: depression, anxiety and other emotions . Is it associated with COVID-19 infection [Single-choice]: :  ○Yes ○No |
| --- |

34. Any previous hypertension, diabetes or other diseases [Single-choice]:

○ Yes _________________ (name of disease)

○ No

Please complete the following table according to your situation

Note: 1 point: mild symptoms; 2 points: definite symptoms, but not affecting life and activities; 3 points: severe symptoms, requiring additional treatment, or already affecting life activities; 4 points: extremely severe symptoms, seriously affecting their lives.

HAMILTON ANXIETY SCALE

| edit  No. | Projects |  | None | light | Medium | Heavy |
| --- | --- | --- | --- | --- | --- | --- |
| 1 | Anxious moods | Worries, anticipation of the worst, fearful anticipation, irritability. | 0 | 1 | 2 | 3 |
| 2 | Tension | Feelings of tension, fatigability, startle response, moved to tears easily, trembling, feelings of restlessness, inability to relax. | 0 | 1 | 2 | 3 |
| 3 | Fears | Of dark, of strangers, of being left alone, of animals, of traffic, of crowds. | 0 | 1 | 2 | 3 |
| 4 | Insomnia | Difficulty in falling asleep, broken sleep, unsatisfying sleep and fatigue on waking, dreams, nightmares, night terrors. | 0 | 1 | 2 | 3 |
| 5 | Intellectual (cognitive) | Difficulty in concentration, poor memory | 0 | 1 | 2 | 3 |
| 6 | Depressed mood | Loss of interest, lack of pleasure in hobbies, depression, early waking, diurual swing. | 0 | 1 | 2 | 3 |
| 7 | Somatic anxiety  (Muscular system) | Pains and aches, twitchings, stiffness, myoclonic jerks, grinding of teeth, unsteady voice, increased muscular tone. | 0 | 1 | 2 | 3 |
| 8 | Somatic anxiety  (Sensory system) | Tinnitus, blurring of vision, hot and cold flushes, feelings of weakness, picking sensation. | 0 | 1 | 2 | 3 |
| 9 | Cardiovascular symptoms | Tachycardia, palpitations, pain in chest, throbbing of vessels, fainting feelings, missing beat. | 0 | 1 | 2 | 3 |
| 10 | Respiratory symptoms | Pressure or constriction in chest, choking feelings, sighing, dyspnea. | 0 | 1 | 2 | 3 |
| 11 | Gastrointestinal symptoms | Difficulty in swallowing, wind, abdominal pain, burning sensations, abdominal fullness, nausea, vomiting, borborygmi, loosness of bowels, loss of weight, constipation. | 0 | 1 | 2 | 3 |
| 12 | Genitourinary symptoms | Frequency of micturition, urgency of micturation, amenorrhea, menorrhagia, development of frigidity, premature ejaculation, loss of libido, impotence. | 0 | 1 | 2 | 3 |
| 13 | Vegetative nervous system symptoms | Dry mouth, flushing, pallor, tendency to sweat, giddiness, tension headache, raising of hair. | 0 | 1 | 2 | 3 |
| 14 | Behavior at interview | Fidgeting, restlessness or pacing, tremor of hands, furrowed brow, strained face, sighing or rapid respiration, facial pallor, swallowing, belching, brisk tendon jerks, dilated pupils, exophthalmos | 0 | 1 | 2 | 3 |
